# Comparative assessment shows the reliability of chloroplast genome assembly using RNA-seq

**DOI:** 10.1038/s41598-018-35654-3

**Published:** 2018-11-27

**Authors:** Carolina Osuna-Mascaró, Rafael Rubio de Casas, Francisco Perfectti

**Affiliations:** 1 Departamento de Genética, Universidad de Granada, Granada, Spain; 2 Unidad de Excelencia “Modeling Nature”, Universidad de Granada, Granada, Spain; 3 Departamento de Ecología, Universidad de Granada, Granada, Spain

## Abstract

Chloroplast genomes (cp genomes) are widely used in comparative genomics, population genetics, and phylogenetic studies. Obtaining chloroplast genomes from RNA-Seq data seems feasible due to the almost full transcription of cpDNA. However, the reliability of chloroplast genomes assembled from RNA-Seq instead of genomic DNA libraries remains to be thoroughly verified. In this study, we assembled chloroplast genomes for three *Erysimum* (Brassicaceae) species from three RNA-Seq replicas and from one genomic library of each species, using a streamlined bioinformatics protocol. We compared these assembled genomes, confirming that assembled cp genomes from RNA-Seq data were highly similar to each other and to those from genomic libraries in terms of overall structure, size, and composition. Although post-transcriptional modifications, such as RNA-editing, may introduce variations in the RNA-seq data, the assembly of cp genomes from RNA-seq appeared to be reliable. Moreover, RNA-Seq assembly was less sensitive to sources of error such as the recovery of nuclear plastid DNAs (NUPTs). Although some precautions should be taken when producing reference genomes in non-model plants, we conclude that assembling cp genomes from RNA-Seq data is a fast, accurate, and reliable strategy.

## Introduction

Chloroplast genomes are an informative and valuable resource for comparative genome evolution, population genetics, and phylogenetic studies^1–3^. Their uni-parental inheritance, low effective population size and stable structure make them extremely useful for studying plant evolution at different taxonomic levels^4–8^. Most plant species have a stable chloroplast genome size ranging from 120 kb to 160 kb^9^, with a highly conserved structure and gene content^10–12^. The typical chloroplast genome structure is quadripartite, comprising two inverted repeats (IRs) separated by a small single copy (SSC) and a large single copy (LSC) region^3,13–16^. Most chloroplast genomes contain 110–130 genes^2^, most of which encode proteins involved in translation and photosynthesis^17^. Several chloroplast genes exhibit conserved flanking regions but internal variability (e.g., *matK* and *rbcL*^18^) and have become basic tools in plant phylogeny and phylogeography^8,19–21^.

The development of high-throughput sequencing technologies has led to a rapid increase in the availability of chloroplast genomes^16,22–24^ making possible the use of complete molecules in phylogenomic analyses^17,25–27^. At present, more than 2,500 complete chloroplast genomes are available^28^. However, the use of complete genome sequencing to obtain reliable chloroplast genomes also poses some caveats and remains relatively expensive. Transcriptome sequencing (RNA-Seq) is comparatively less complex because it yields only the sections of the genome that are transcribed into RNA, providing a relatively cheap and fast method to obtain large amounts of functional genomic data^29–32^. Accordingly, global initiatives such as the 1,000 plants (1KP) project have generated a wealth of transcriptomic data for over more than 1,000 plant species^33^. Since the chloroplast genome appears to be fully transcribed, RNA-Seq data could potentially be used to obtain the complete chloroplast genome^34^. However, the reliability of assembling chloroplast genomes from transcriptomic versus genomic data has not been thoroughly evaluated.

In this study, we compared the reliability of RNA-Seq to genomic DNA libraries to obtain cpDNA complete sequence. For this purpose, we assembled for the first time the complete chloroplast genome of three *Erysimum* (Brassicaceae) species: *Erysimum mediohispanicum*, *E*. *nevadense*, and *E*. *baeticum* from genomic libraries. *Erysimum* constitutes an interesting case study because it is a genus that encompasses wide diversity attained through rapid and complex evolutionary processes^35–37^, while being evolutionarily close enough to *Arabidopsis thaliana* to render the use of genomic and transcriptomic references from this model species relatively easy. We assembled the chloroplast genomes of these three species using different computational approaches and compared several genetic features (gene content, presence of repeats, microsatellites –SSRs–, etc) across genomes obtained RNA-Seq or genomic DNA. Based on these results, we assessed a) The characteristics of the chloroplast genomes of *Erysimum* spp.; b) the genomic coverage provided by RNA-Seq across species and c) a bioinformatic approach to ensure reliable chloroplast genome assembly from transcriptomic data. In the light of these results, we propose a pipeline-like methodology for processing RNA-Seq reads into high-quality cp genomes.

## Material and Methods

### Plant materials

Fresh leaves and flower buds of *Erysimum mediohispanicum*, *E*. *nevadense*, and *E*. *baeticum* were collected from several populations located in the Baetic Mountains, South of Spain (Table 1 shows the code and location of all populations). Leaves were dried and preserved in silica gel until DNA extraction. Pre-opening flower buds at the same development stage were stored in liquid nitrogen for RNA extraction.

**Table 1:** Details of the plant populations sampled: Taxon, population code, sampled tissue, location, and geographical coordinates.

Taxon	Population code	Sample	Location	Elevation	Geographical coordinates
*E*. *baeticum*	Ebb09	Leaves	Sierra Nevada, Almería, Spain	2128	37°05′46″N 3°01′01″W
	Ebb07	Buds	Sierra Nevada, Almería, Spain	2128	37°05′46″N 3° 01′01″W
	Ebb10	Buds	Sierra Nevada, Almería, Spain	2140	37°05′32″N 3° 00′ 40″ W
	Ebb12	Buds	Sierra Nevada, Almería, Spain	2264	37°05′51″N 2°58′06″W
*E*. *mediohispanicum*	Em21	Leaves and buds	Sierra Nevada, Granada, Spain	1723	37° 08′ 04″N 3°25′43″W
	Em71	Buds	Sierra de Huétor, Granada, Spain	1352	37°57′10″N 2°29′24″W
	Em39	Buds	Sierra Jureña, Granada, Spain	1272	37°19′08″N 3°33′11″W
*E*. *nevadense*	En14	Leaves	Nigüelas, Granada, Spain	2314	37°01′27″N 3°28′08″W
	En12	Buds	Sierra Nevada, Granada, Spain	2255	37°05′37″N 2°56′19″W
	En10	Buds	Sierra Nevada, Granada, Spain	2321	37°06′37″N 3°24′18″W
	En05	Buds	Sierra Nevada, Granada, Spain	2074	37°06′35″N 3°01′32″W

### DNA extraction and sequencing

We used an individual sample for each species (Table 1). For each sample at least 60 mg of leafs was disrupted using a Beadbug microtube homogenizer (Benchmark Scientific, Edison, NJ) with 2 mm steel beads. Total genomic DNA was isolated using the GenElute Plant Genomic DNA Miniprep kit (Sigma-Aldrich, St. Louis, MO) following the manufacturer’s protocol. The quantity and the quality of the obtained DNA were checked using a NanoDrop 2000 spectrophotometer (Thermo Fisher Scientific, Wilmington, DE, United States), and the integrity of the extracted genomic DNA was checked on agarose gel electrophoresis. Isolated DNA was sent to Macrogen (Macrogen Inc., Seoul, South Korea) to perform library preparation and sequencing. Library preparation for deep sequencing was carried out using the TruSeq Nano DNA Library Preparation Kit (350 bp insert size). The sequencing of the three cDNA libraries (*E*.*mediohispanicum*, *E*.*nevadense*, and *E*.*baeticum*) was carried out using the Illumina HiSeq X platform and following the paired-end 150 bp strategy. A summary of sequencing statistics is shown in Table S1 (Supporting Information).

### RNA extraction and sequencing

For each population, three replicas consisting of one pre-opening bud each were used. They were snap-frozen in liquid nitrogen and disrupted with a mortar. Total RNA was isolated using the Qiagen RNeasy Plant Mini Kit following the manufacturer’s protocol. The quality and quantity of the RNA obtained was checked using a NanoDrop 2000 spectrophotometer (Thermo Fisher Scientific, Wilmington, DE, United States), and analyzed with the Agilent 2100 Bioanalyzer system (Agilent Technologies Inc). The RNA was sent to Macrogen (Macrogen Inc., Seoul, South Korea) for library preparation and sequencing. We used a rRNA-depletion protocol (Ribo-Zero^38^) to perform a mRNA enrichment and to avoid sequencing rRNAs. Library preparation was performed using the TruSeq Stranded Total RNA LT Sample Preparation Kit (Plant). The sequencing of the 9 libraries was carried out using the Hiseq 3000–4000 sequencing protocol and TruSeq 3000–4000 SBS Kit v3 reagent, following a paired-end 150 bp strategy on the Illumina HiSeq 4000 platform. A summary of sequencing statistics is shown in Table S1 (Supporting Information).

### Chloroplast genome assembly and annotation

**Figure 1:**
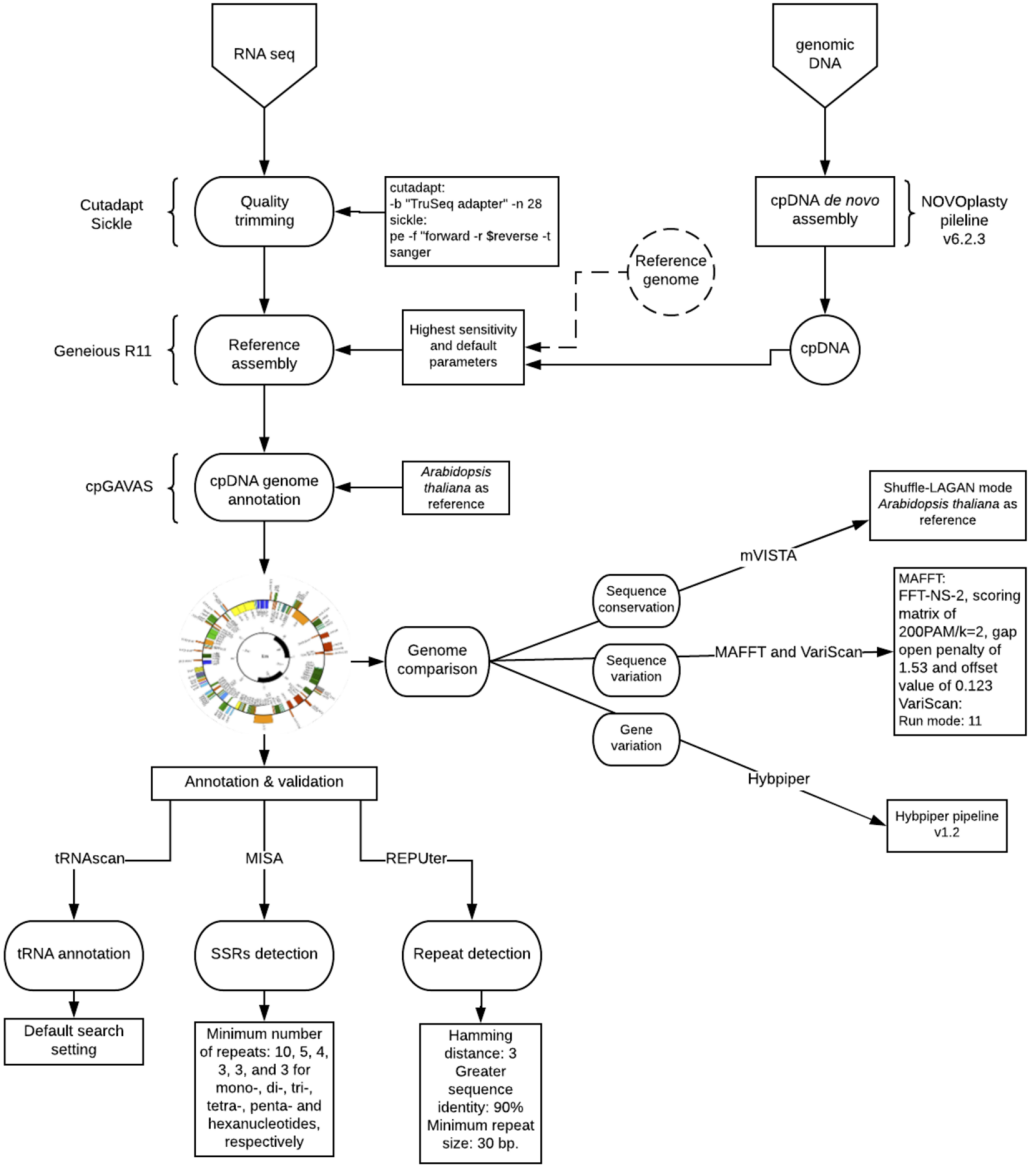
A flow chart depicting the bioinformatics analyses to assembly cp genomes.

We assembled *de nov*o the chloroplast genomes of *E*. *mediohispanicum*, *E*. *nevadense*, and *E*. *baeticum* using the NOVOPlasty pipeline v.6.2.3^7^ (Fig. 1). Basically, through this pipeline a cp genome is assembled from whole genome sequencing (WGS) data, starting from a related single seed sequence iteratively extended bidirectionally until the circular genome is obtained. We used untrimmed reads as recommended by Dierckxsens *et al*.^7^ and *Arabidopsis thaliana* cpDNA sequence (NC_000932.1) as the seed, since *Erysimum* is a close relative of *Arabidopsis*^39^. We specified the following parameters: automatic insert size detection, a genome range from 120000 to 200000, a K-mer value of 39, an insert range of 1.6, a strict insert range of 1.2, and the paired-end reads option.

After assembling the full chloroplast genome of *E*. *medihospanicum*, we proceeded to assemble the cp genomes from the RNA-Seq data by using this chloroplast genome as a reference. From the RNA-Seq libraries, we first trimmed the adapters in the raw reads using cutadapt v.1.15^40^. For trimming adapters in 5′ and 3′ direction we used the “-b” option, and only used the prefix of the adapter sequence that is common to all “TruSeq Indexed Adapter” sequences (AGATCGGAAGAGCACACGTCTGAACTCCAGTCAC). In addition, we used the “-n” option to search repeatedly for the adapter sequences (28 iterations). This option ensures that the correct adapters are detected by searching in loops until any adapter match is found or until the specified number of rounds is reached. Then, we quality-filtered the reads using Sickle v.1.33^41^, a trimming software which uses sliding-window analyses along with quality and length thresholds to cut and discard the reads which do not fit the selected threshold values. We specified the “pe” option for paired-end reads and the “-t” to use Illumina quality values (see https://github.com/najoshi/sickle). After filtering, we used the read mapper of Geneious R. 11^42^ with the highest sensitivity and default parameters (http://www.geneious.com)^42^ for a reference-guided assembly of the trimmed reads using the *E*. *mediohispanicum* reference assembly (see above). We validated the results obtained with Geneious by comparing them to read maps obtained with the BWA read mapper^43^.

The program cpGAVAS (Chloroplast Genome Annotation, Visualization, Analysis and GenBank Submission Tool^44^) was used for annotating and visualizing the cp genomes. This program takes as input a FASTA file containing the genome information and performs bioinformatic analyses to annotate the genome. We used the annotated *Arabidopsis thaliana* cp genome (NC_000932.1)^45^. Protein coding genes were manually curated. Lastly, cpGAVAS gives as output the statistics of the annotation process, the annotated genome, and a visualization of the annotated genome. The annotations were then manually curated using Geneious R.11^42^. All transfer RNA sequences (tRNA) encoded in the cp genomes were verified using tRNAscan-SE 2.0^46^ with the default search settings. The step-by-step process is presented in Fig. 1.

### Comparative analysis among cp genomes assemblies

To compare the cp genomes assembled from DNA or RNA libraries, we used the mVISTA software, part of the VISTA suite of tools for comparative genomics (http://genome.lbl.gov/vista/mvista/submit.html). This software compares DNA sequences from different species by pairwise alignment and allows the visualization of these alignments with annotation information. The output allows the identification of homologies between sequences, determining the percentage of identity between them using a sliding window of predefined length. We selected default parameters, a RankVISTA probability threshold of 0.5, and the Shuffle-LAGAN mode, which is a global alignment algorithm for finding rearrangements (inversions, transpositions, and some duplications). We used the *A*. *thaliana* cpDNA as a reference (NC_000932.1)^45^. The sequence conservation profiles were visualized in mVISTA plots^47^.

We investigated the degree of within-genome variation of the assembled cp genomes. In particular, we performed a reference-guided assembly in which we remapped the quality-trimmed reads (as for the RNA-Seq assemblies, see above) to each assembled genome using the Geneious R. 11^42^ mapper with medium-low sensitivity and default parameters (http://www.geneious.com)^42^. Later, we estimated the percentage of pairwise identity of each assembly. This statistic gives the average identity (as %), computed by scoring a hit when all pairs of bases are identical and dividing it by the total numbers of pairs.

For each species, we explored the degree of overall sequence variation found within the three replicas of RNA-Seq assembled genomes and then compared the results to those of a similar analyses that included also the genome assembled from genomic libraries. For this purpose, we estimated the nucleotide diversity (π) among the three replicas of cp genomes assembled from RNA-Seq, and then computed it again including the corresponding genomic library. Genomes were first aligned using MAFFT with the following parameters: FFT-NS-2 fast progressive method algorithm, a scoring matrix of 200PAM/k = 2, gap open penalty of 1.53 and offset value of 0.123. Then, we estimated the cpDNA nucleotide diversity using VariScan v.2.0.3^48^.

We studied the degree of sequence variation of some relevant chloroplast genes within the three replicas of RNA-Seq, and then explored it but including the genes assembled from genomic libraries. We first extracted and assembled all the chloroplast genes using the HybPiper pipeline v.1.2^49^. This pipeline uses BWA^43^ to align reads to target sequences, and SPAdes^50^ to assemble these reads into contigs. Once cpDNA genes were obtained, we selected 12 genes out of the total: *rbcl*, *psaA*, *psbA*, *ndhK*, *atpA*, *atpH* (with an important function in the photosynthesis process^51^),* rpoA*,* rps3*,* rrn16S*,* trnH* (as self replication genes^52^), *yfc2* (the largest plastid gene in angiosperms^53^), and *matK* (the only maturase of higher plants and widely used in angiosperm systematic^54^). Then, we aligned these genes using MAFFT, as explained above. Lastly, we calculated the percentage of pairwise identity between the genes obtained from the three RNA-Seq replicas, and the same but including those from genomic libraries.

The size and location of repeat sequences, including palindromic, reverse and direct repeats, within these cp genomes were identified using REPuter software^55^. Following Asaf *et al*.^8^ and Ni *et al*.^56^ REPuter was parametrized with the following settings: Hamming distance of 3; 90% or greater sequence identity; and minimum repeat size of 30 bp.

Simple sequence repeat (SSR) elements were detected using the Perl script MISA^57^ by setting the minimum number of repeats to 10, 5, 4, 3, 3, and 3 for mono-, di-, tri-, tetra-, penta- and hexanucleotides, respectively.

### Analysis of minimum transcriptome depth to produce quality cp genomes assemblies

To analyze the impact that sequencing depth has in the assemblage of transcriptome data into a complete cp genome, we subsampled the transcriptome reads of *E*. *nevadense* four times at 1 M, 5 M, 10 M, 20 M and 30 M paired reads. These reads were processed and mapped to the cpDNA of *E*. *mediohispanicum* with Geneious R.11^42^ with medium-low sensitivity and default parameters as previously done. We calculated several mapping quality indexes (coverage of bases, expected errors, mean confidence, and % of Q40 positions) with Geneious R.11^42^ and plotted them against the sub-sampling depth.

### Cross-validation of the methodology

In order to estimate the recovery of complete cpDNA chromosomes from RNA-Seq libraries in other plant species, we downloaded five transcriptomes from the Sequence Read Archive website and processed them with our workflow. We downloaded two *A*. *thaliana* (SRR6757372; SRR6676021), one *E*. *cheiri* (SRR5195368), one *Moricandia suffruticosa* (SRR4296233), one *M*. *arvensis* (SRR4296231), one *Oriza sativa* (SRR7079258), and one *Zea mays* (ERR1407273) transcriptome. These libraries were trimmed and quality filtered using cutadapt v.1.15^40^ and Sickle v.1.33^41^ with the same parameters described above, and mapped using Geneious R.11^42^ to cp genomes of the same species (or the closest relative available): genbank accession NC_000932 for *A*. *thaliana*, our *E*. *mediohispanicum* cp genome for the *E*. *cheiri* sample, *Brassica napus* GQ861354 for the *Moricandia* samples, *Oriza sativa* NC_001320 for *O*. *Sativa*, and *Z*. *mays* NC_001666 for *Z*. *mays*.

## Results

### Chloroplast genome assembly and annotation

#### Genomic DNA libraries

**Figure 2:**
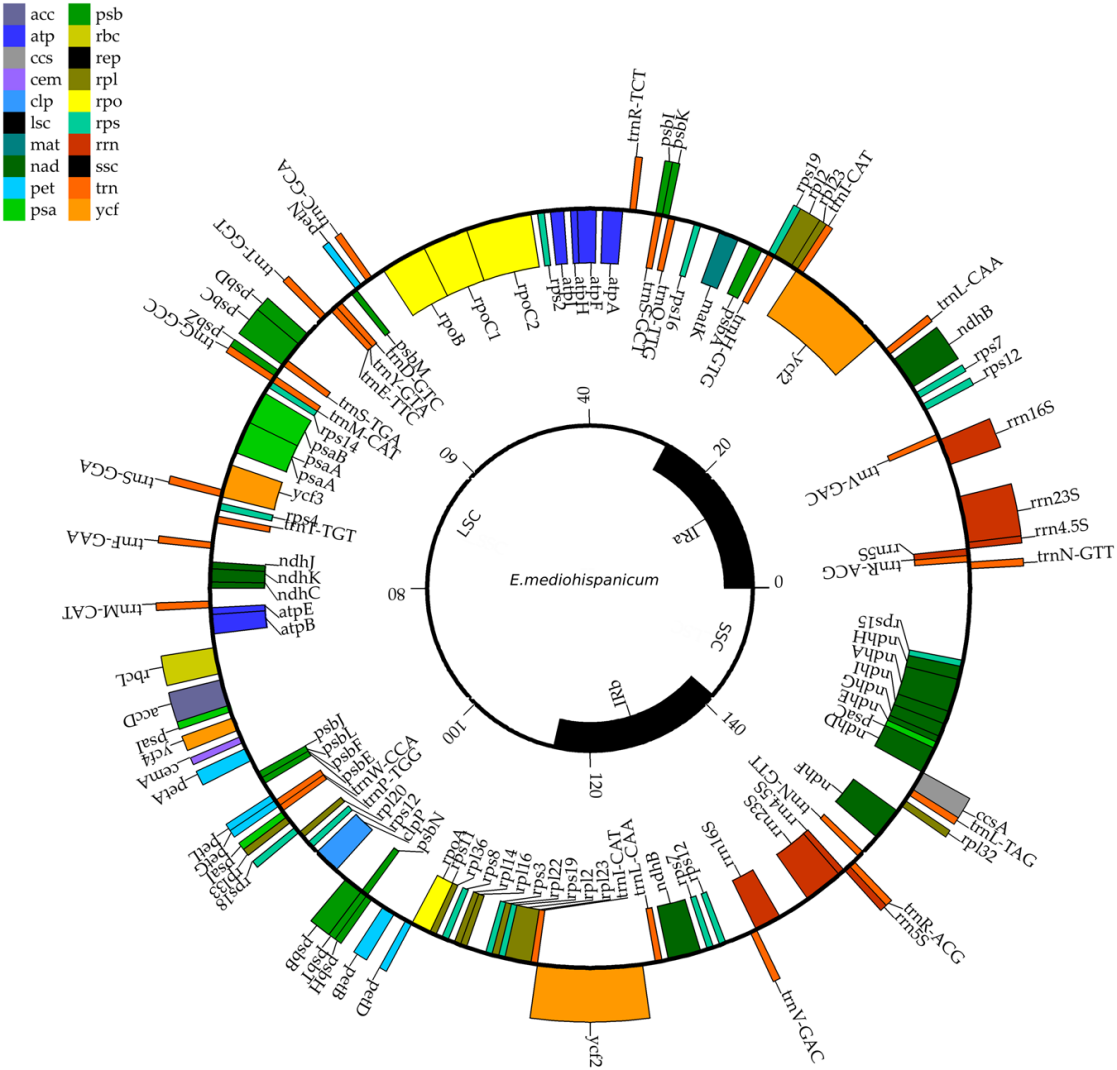
Chloroplast genome map of *Erysimum mediohispanicum*. Genes drawn inside the circle are transcribed clockwise, and those outside are counter-clockwise. Genes belonging to a different functional group are shown in different colors. See supplementary material for functional category of these genes.

**Figure 3:**
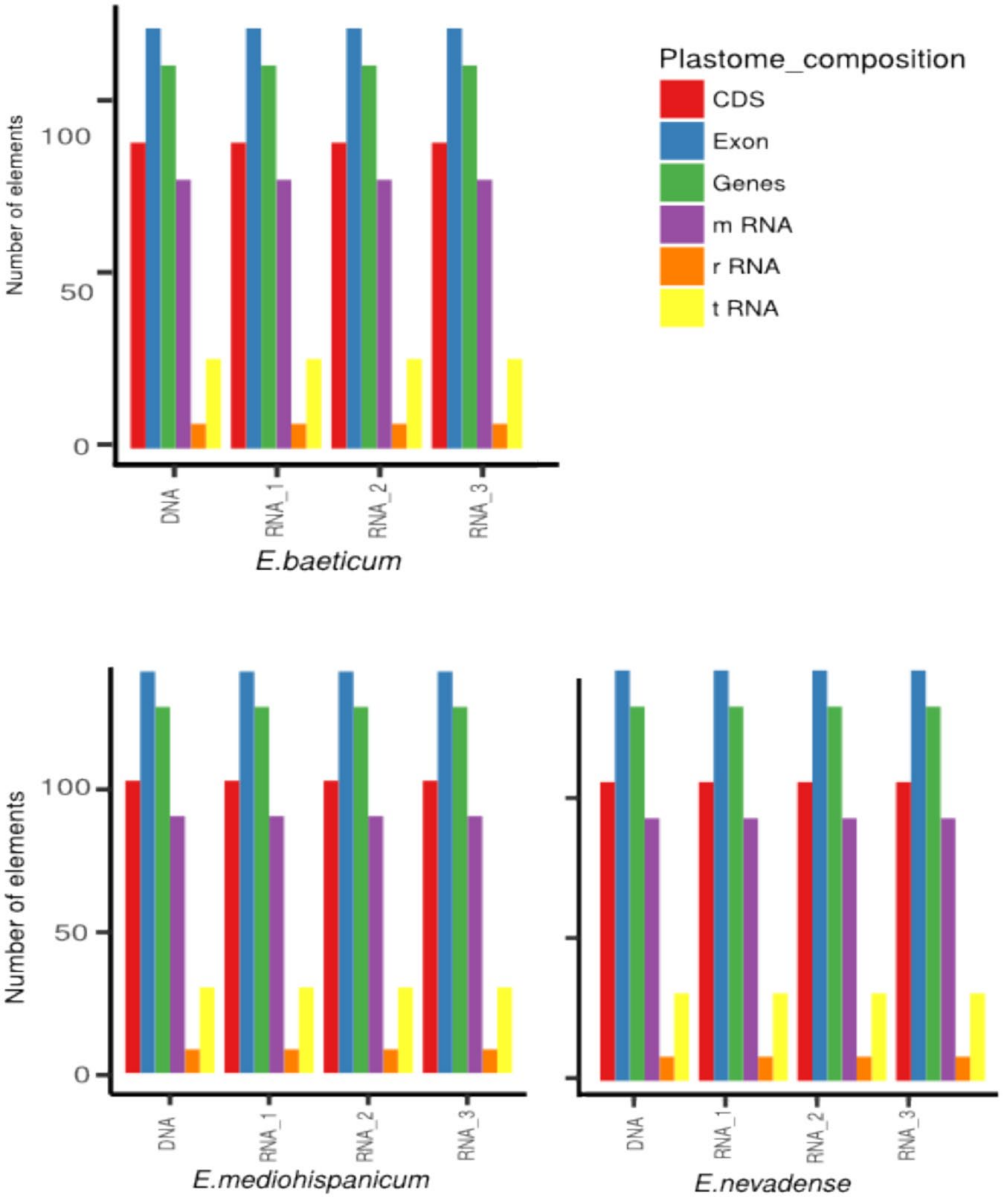
Composition of *Erysimum baeticum*, *E*. *mediohispanicum*, *and E*. *nevadense* cp genomes, obtained from genomic data and for the three RNA-Seq replicas.

We assembled *de novo* the whole chloroplast genomes of three *Erysimum* species. The assembled genomes were circular and have a total length of 154,599 bp, 154,660 bp, and 154,581 bp in *E*. *mediohispanicum*, *E*. *nevadense*, and *E*. *baeticum*, respectively (Figs 2, S1 and S2). These chloroplast genomes displayed the typical quadripartite structure of most angiosperms (See Table 2), comprising a pair of inverted repeats (IRs; 26,429 bp, 26,442 bp, and 26,429 bp respectively), the large single copy region (LSC; 136,628 bp, 136,724 bp, and 136,625 bp respectively), and the small single copy region (SSC; 83,853 bp, 83,804 bp, and 83, 767 respectively). The gene content of the three chloroplast genomes was very conserved (Table S2). Thus, the number of unique protein-coding genes was 124 for the three species. These chloroplast genomes contained 29 unique transfer RNA genes and 8 unique ribosomal RNA genes (Fig. 3). The number of intra-gene regions was 150 in each cp genome. We found eight split genes (*rpl2*, *atpF*, *rpoC1*, *psaA*, *ycf3*, *clpP*, *ndhB*, *ndhA*; see Table 3) with intronic regions for each cp genome. Lengths of intronic regions are shown in Table S3. The overall GC content was 36.6%, indicating similar conserved GC levels among the *Erysimum* chloroplast genomes. A summary of the number of sequences assembled, mean assembly coverage, and percentages of pairwise identity are shown in Table S4 (Supporting Information).

**Table 2:** Characteristics of the chloroplast genomes of *Erysimum*: type of library (genomic DNA or RNA-Seq library), length of the cp genome (bp), number of assembled reads, length of the two inverted repeats (IRa and the IRb), length of the small single copy (SSC), and of the large single copy (LSC) region, and GC% content.

Taxon	Population code	Type of library	Lenght (bp)	Assembled reads	IRa (bp)	SSC (bp)	IRb (bp)	LSC (bp)	GC %
*E*. *baeticum*	Ebb09	Genomic DNA	154,581	983,811	26,429	83,767	26,426	136,625	36.6
	Ebb07	RNA-Seq libraries	154,791	3,727,511	25,783	95,135	13,797	134,715	37.5
	Ebb10	RNA-Seq libraries	154,768	9,963,413	25,847	95,396	14,419	135,662	36.5
	Ebb12	RNA-Seq libraries	154,761	10,356,264	24,617	95,167	13,305	133,089	36.5
*E*. *mediohispanicum*	Em21	Genomic DNA	154,599	1,414,714	26,429	83,853	26,429	136,628	36.6
	Em71	RNA-Seq libraries	154,788	1,314,441	24,671	95,187	13,303	133,161	36.5
	Em39	RNA-Seq libraries	154,827	13,595,017	26,472	83,764	24,099	134,335	36.5
	Em21	RNA-Seq libraries	154,251	19,075,780	25,280	89,248	18,133	132,661	36.6
*E*. *nevadense*	En14	Genomic DNA	154,660	1,554,542	26,442	83,840	26,442	136,724	36.6
	En05	RNA-Seq libraries	153,467	12,482,406	25,863	85,139	24,831	135,833	36.7
	En10	RNA-Seq libraries	154,834	9,515,436	25,902	85,182	23,492	134,576	36.7
	En12	RNA-Seq libraries	154,747	5,338,711	25,764	84,289	24,027	134,080	36.7

**Table 3:** Comparison of RNA-Seq vs. genomic assembly of chloroplast genomes. Number of protein-coding genes, tRNA, mRNA, rRNA, exons, coding sequences (CDS), genes with introns, repeat sequences, and the total number of repeats (i.e. including forward, reverse, palindrome, and complemented repeats) in different chloroplast regions (IRa, SSC, IRb, and LSC) for chloroplast genomes obtained from genomic DNA and chloroplast genomes obtained from RNA-Seq libraries are presented. The eight genes showing introns were *rpl2*, *atpF*, *rpoC1*, *psaA*, *ycf3*, *clpP*, *ndhB*, and *ndhA*.

Taxon	Population code	Type of library	Protein-coding genes	tRNA	mRNA	rRNA	Exons	CDS	Genes with introns	Total repeats number	Forward repeats	Reverse repeats	Palindrome repeats	Complemented repeats	Total repeats in IRa region	Total repeats in SSC region	Total repeats in IRb region	Total repeats in LSC region
*E*. *baeticum*	Ebb09	Genomic DNA	124	29	87	8	136	99	8	49	31	0	18	0	7	33	7	2
	Ebb07	RNA-Seq	124	29	87	8	136	99	8	50	25	0	25	0	7	36	7	0
	Ebb10	RNA-Seq	124	29	87	8	136	99	8	61	36	0	23	2	8	45	8	0
	Ebb12	RNA-Seq	124	29	87	8	136	99	8	64	36	0	26	2	8	48	8	0
*E*. *mediohis-panicum*	Em21	Genomic DNA	124	29	86	8	135	98	8	63	37	0	24	2	10	40	3	10
	Em71	RNA-Seq	124	29	87	8	135	98	8	60	36	0	22	2	7	46	7	0
	Em39	RNA-Seq	124	29	87	8	135	98	8	74	36	0	36	2	13	48	13	0
	Em21	RNA-Seq	124	29	87	8	135	98	8	69	42	1	24	2	9	50	9	1
*E*. *nevadense*	En14	Genomic DNA	124	29	87	8	136	99	8	78	51	1	25	1	7	59	7	5
	En05	RNA-Seq	124	29	86	8	136	99	8	73	38	0	33	2	11	51	11	0
	En10	RNA-Seq	124	29	87	8	136	99	8	70	36	0	31	2	11	48	11	0
	En12	RNA-Seq	124	29	87	8	136	99	8	69	36	0	31	2	9	49	9	2

#### RNA-Seq libraries

We assembled the chloroplast genomes from three different replicas for each of the three species. We recovered high-quality complete chloroplast genomes that were very similar to those obtained from genomic DNA. In particular, for *E*. *mediohispanicum* the retrieved chloroplast genome sizes were 154,788 bp, 154,827 bp, and 154, 251 bp; for *E*. *nevadense* genome sizes were 153,467 bp, 154,834 bp, and 154,747 bp; and for *E*. *baeticum* were 154,791 bp, 154,768 bp, and 154,761 bp. The IR, LSC, and SSC contents (See Table 2), as well as the protein-coding gene contents, tRNAs, and rRNAs were very similar between species replicates but when comparing with the chloroplast genomes obtained from genomic DNA, we found that the IRb regions were shorter and SSC regions were slightly larger (see Fig. S3 for chloroplast borders comparison). We found the same eight split genes with introns regions that were found in cp genomes obtained from genomic DNA (*rpl2*, *atpF*, *rpoC1*, *psaA*, *ycf3*, *clpP*, *ndhB*, *ndhA*; see Table 3). The lengths of all intronic regions are shown in Table S3. We found the same number of intra-gene regions using RNA-seq and genomic libraries (150 in each cp genome). The overall GC was 36%. A summary of assembly statistics including the bases assembled, mean assembly coverage, and percentages of pairwise identity are shown in Table S4. The results of the mapping assembly using BWA were highly similar (Table S5).

### Repeat and SSRs analyses

**Figure 4:**
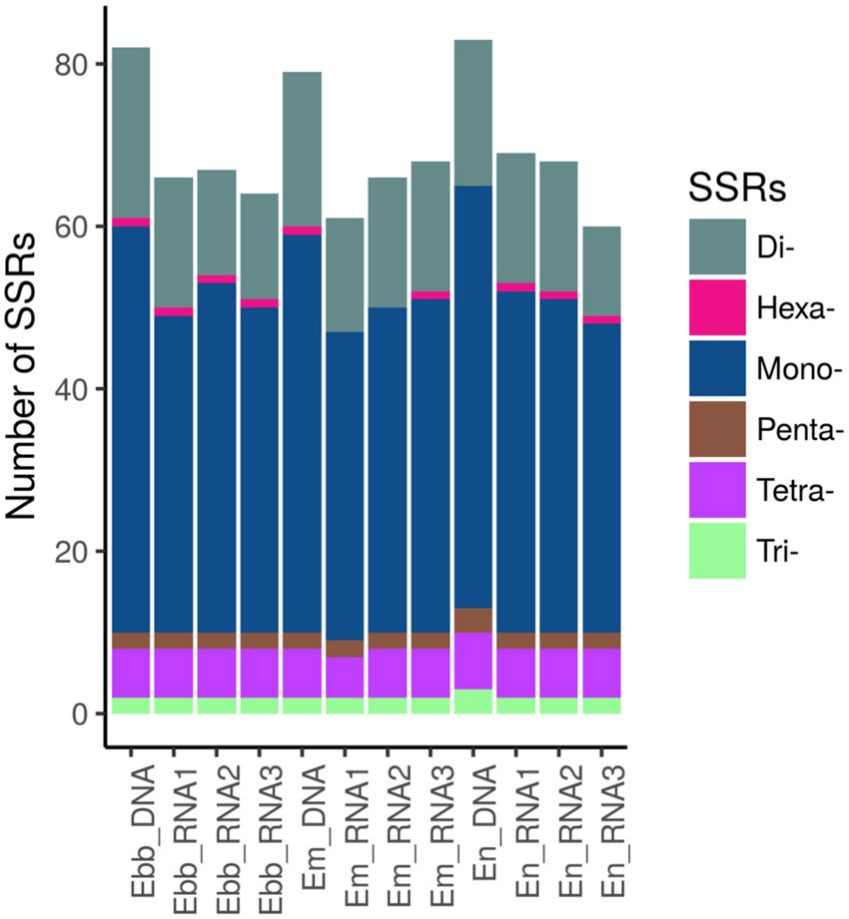
The number of single small repeats (SSRs) sequences in the chloroplast genomes of *Erysimum* species, obtained from genomic data and for the three RNA-Seq replicas.

The total number of repeats was 64, 78, and 65 in *E*. *mediohispanicum*, *E*. *nevadens*e, and *E*. *baeticum*, respectively. Forward repeats were the most common across the three species, followed by palindromic repeats. Reverse and complement repeats were found in low abundance (Table 3). In particular, *E*. *mediohispanicum* contained 38 forward, 24 palindromic, and two complement repeats; *E*. *nevadens*e contained 51 forward, 25 palindromic, and one complement repeats; and *E*. *baeticum* contained 38 forward, 25 palindromic, and two complement repeats, respectively. In addition, the repeats from the three species had a sequence identity greater than 90%. The length of these repeats ranged for all the species from 30 to 26,429 bp, and the most common copy length had 30 bp. The number of repeats in the chloroplast genome assembled from RNA-Seq data was similar to that obtained from genomic DNA (Table 3). The average number of repeats was 78.3, 90, and 84, for the three replicas of *E*. *mediohispanicum*, *E*. *nevadense*, *and E*. *baeticum*, respectively. Forward repeats were the most common, followed by palindrome repeats, with lower levels of reverse and complemented repeats. The repeats of these population samples had a sequence identity greater than 90% for each species. The length of these repeats reached from 30 to 14, 353 bp, with the units with 30 bp being also the most common. The SSRs contained in the three chloroplast genomes were analyzed using the MISA Perl script (Fig. 4). The number of detected SSRs were 78, 83, and 81, for *E*. *mediohispanicum*, *E*. *nevadense*, and *E*. *baeticum*, respectively. Among them, most of the SSRs were mononucleotide repeats, followed by dinucleotide and tetranucleotide repeats. The hexanucleotides were the less frequent type. Among these SSRs, mononucleotide A/T repeat units were the most represented, with a proportion of 58% in *E*. *mediohispanicum*, 59% in *E*. *nevadense a*nd 59% in *E*. *baeticum*. The number of SSRs identified in cp genomes assembled from RNA-Seq was lower than the number identified in cp genomes obtained from genomic libraries. We found a total of 61, 66, and 68 SSRs in the each of the three *E*. *mediohispanicum* population samples; 68, 67, and 68 in the three *E*. *baeticum* samples, and 69, 68, and 60, in the three *E*. *nevandense* samples. Table S6 shows the numbers of SSR’s that were quantitatively different between cp genomes assembled from genomic and RNA-Seq libraries. Among them, most of the SSRs were also mononucleotide repeats, with A/T repeats showing the highest proportion in the three replicas per species.

### Genomic comparison

**Figure 5:**
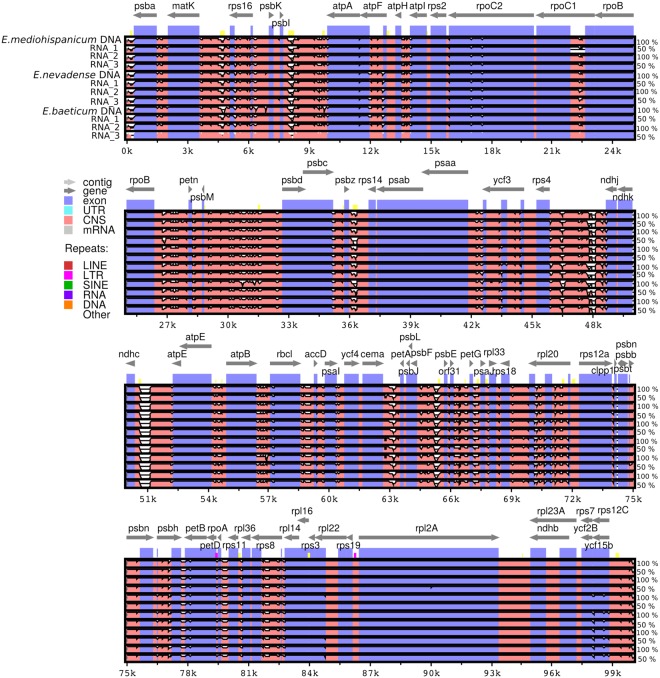
Sequence identity plots among the *Erysimum* chloroplast genomes, with *Arabidopsis thaliana* as a reference. Annotated genes are displayed on the top. A cut-off of 50% identity was used for the plot. The vertical scale represents the percent identity between 50 and 100%. Genome regions are color-coded as CNS (conserved non-coding sequences), exons, and introns. The color legend is summarized in the upper left-hand corner.

Results from mVISTA plots revealed a high similarity, with 99% of shared sequence identity in pairwise comparisons, between chloroplast genomes from genomic libraries and those from RNA-Seq libraries (See Fig. 5; the top and bottom percentage bounds are shown to the right of every row). These plots also showed a high degree of synteny between the three *Erysimum* species. In addition, the two IR regions were more similar than the LSC and SSC regions in all these species. Lastly, non-coding regions reveal a higher divergence than coding regions. Nucleotide diversity (π) was lower among the three replicas assembled from RNA-Seq. In contrast, nucleotide diversity increased dramatically (~ three orders of magnitude) when including the cp genome from genomic libraries in the alignments (0.35988 vs. 0.00008 for *E*. *mediohispanicum;* 0.36617 vs. 0.00037 for *E*. *nevadense*; and 0.36068 vs. 0.00123 for *E*. *baeticum*). Percentages of pairwise identity were always higher than 99% when comparing genes assembled from the different RNA-Seq replicas, and this similarity did not decrease when including genes assembled from genomic libraries (see Table S7).

### Effect of sequencing depth

We assembled the chloroplast genomes from four different resampling of an *E*. *nevadense* transcriptome at 1 M, 5 M, 10 M, 20 M and 30 M paired reads. With this particular transcriptome, chloroplast genomes were obtained with coverage >95% from libraries of only 1 M reads, and with coverage >99% from sequencing depths >5 M reads (see supplementary information Figure S4). As expected, all metrics of mapping quality as well as the mean coverage at each position (p < 0.0001; R^2^ = 0.921; from ~170 K to close to 200 K) increased significantly with sequencing depth (see supplementary information Figure S4).

### Cross-validation results

We assembled the cp genomes from RNA-Seq data of five species: *A*. *thaliana*, *E*. *cheiri*, *M*. *suffruticosa*, *M*. *arvensis*, *O*. *sativa*, and *Z*. *mays*. All estimated parameters (assembly consensus length, confidence mean, Q20, Q30 and Q40 sequence quality scores, total number of assembled reads, percentage of pairwise identity, mean coverage, and coverage with respect to the reference sequence) showed that assembling cpDNA from RNA-Seq data was feasible, albeit the reliability of the assembly was dependent on the RNA-Seq reads used (see Table S8).

## Discussion

Our results showed that complete chloroplast genomes can be reliably assembled from transcriptomic data. We studied some *Erysimum* species as a proof-of-concept, and obtained genomes congruent in structure and sequence with previously published chloroplast genomes^2,3,58^. Both the chloroplast genomes assembled from transcriptomic and genomic libraries exhibit the typical quadripartite structure, low GC content, and are mainly composed of polythymine (polyT), and polyadenine (polyA) repeats^59^. Chloroplast genomes assembled from RNA-Seq data are highly similar in terms of SSRs, number of repeats, and plastome composition (CDS, exons, genes, rRNA, and tRNA) to those assembled from genomic libraries. Moreover, the similarity of the genomic and RNA-Seq assemblages validates that chloroplast genomes are fully transcribed. This is in line with findings from Shi *et al*.^34^ who showed full transcription of chloroplast genome in photosynthetic eukaryotes using several tissues (flowers, complete seedlings, and seedlings shoots). Here, we show that chloroplast genomes of flower buds, the tissues we have used to obtain the RNA-Seq libraries, are also fully transcribed. Therefore, chloroplasts appear to be fully transcribed across organs and development stages in angiosperms, at least in samples containing functional plastids.

We have found significant differences in nucleotide diversity when comparing both kinds of assemblages (RNA-Seq vs genomic libraries). This may be explained by post-transcriptional modifications, i.e., by RNA-editing^60^. However, we found that nucleotide diversity greatly increased when including the assemblies from genomic libraries into the alignments. Accordingly, nucleotide diversity was lower when only comparing the three replicates of the RNA-Seq data. This implies that genomic assemblies were more heterogeneous or noisier than transcriptomic ones. Since both libraries were obtained using similar Illumina platforms, it appears that the genomic libraries were intrinsically more heterogeneous. This heterogeneity is likely caused by segments of chloroplast DNA transferred to the nuclear genome (i.e., nuclear plastic DNA or NUPT) that may potentially be incorporated during the mapping procedure introducing heterogeneity (i.e., within-genome polymorphism) into the cpDNA genomic assemblies^45,61^. However, NUPTs are generally fragmented and eliminated from the nuclear genome and therefore not transcribed, or transcribed at low level^62–64^, and therefore they should not be recovered in the RNA-Seq libraries. Moreover, the lack of differences in pairwise identity when only comparing genes from RNA-Seq to those from genomic libraries may be consequence of NUPTs located at the intergenic regions, as have been found in previous studies (e.g., only 25% of NUPTs in *Arabidopsis thaliana* are located in genes^65^). NUPTs are well documented in plants^66^, and they often represent a significant part of the nuclear genome^67,68^. Because of the maternal inheritance in most plant genera^69^, cpDNA is widely used for the inference of relationship among plants. Therefore, the presence of NUPT into cp genomes may lead to erroneous phylogenetic inferences^66^. According to our results, using cpDNA assembled from transcriptomes might reduce the problems due to NUPT inclusion when using cpDNA in phylogenomics. Alternatively, methods specifically designed to correct these assembly errors have been developed for genomic data, such as the dnaLCW method^61^, and should be consider whenever possible. However, validating that NUPTs are a source of error in cp genome assembly requires comparison with a reference genome, which is currently not available for *Erysimum*. Therefore, the potential misleading mapping caused by this type of genetic elements will require further studies.

When we tested our methodology across several plant species, we found that assembling chloroplast genomes from RNA-Seq data is a relatively fast and flexible approach. In the light of these results, we put forward a pipeline-like procedure in the hope that it can be useful to other researchers (Fig. 1). In addition, we showed that, although the chloroplast genome coverage increased with the number of reads used for the assembly, 1 M reads was sufficient to obtain a 95% coverage of the cp genome. These results corroborate that the chloroplast could be fully transcribed and is easily assembled from transcriptomic data even at low-medium coverage. Moreover, cross-validation (Supplementary Table S8) showed that assembling the cp genome using transcriptomes from the SRA database is feasible even though the reliability of the assemblage is always a function of the tissue and methodology used. For example, *Arabidopsis thaliana* cp genomes, assembled from RNA-Seq data coming from different libraries (SRR667021 and SRR6757372), produced different assembly results that were related to differences in coverage and number of reads. Furthermore, the genome of *E*. *cheiri* cpDNA was surprisingly not fully assembled despite being a closely related species to the *Erysimum* species used in this study^37^. However, this result may be explained by the fact that this *E*. *cheiri* transcriptome was obtained from petals. The reliability of our results is probably attributable to careful sample preparation (our RNA-Seq samples were submitted to a treatment that depleted rRNA implying that the samples were enriched in the other types of RNA), and because sequencing depth, at least over a minimum threshold ~5 M reads (see Figure S4), does not appear to be a crucial factor. Therefore, as a general rule, samples obtained from photosynthetic tissues, depleted in rRNA and high quality sequenced (as indicated by quality scores) are likely to be trustworthy.

We conclude that assembling cp genomes from good quality transcriptomic data (either obtained *de novo* or downloaded from public databases such as the SRA database) may be a straightforward approach in plant systematics and phylogeny. In fact, this approach may reduce the risk of incorporating NUPTs avoiding posterior phylogenetic incongruences, although precautions must be taken due to the possibility of RNA editing, and alternative methods^61^ could be also used to minimize the assembly of NUPTs, or other nuclear DNA, into cp genomes. In summary, we think the pipeline presented here is an accessible and time saving approach to produce high-quality cp genomes that could complement other genomic approaches.

## Electronic supplementary material

Supplementary Material

## Data Availability

Chloroplast genomes were submitted to GenBank with the following accession numbers: *E*. *baeticum*: Ebb07 (MH414570), Ebb09 (MH414571), Ebb10 (MH414572), Ebb12 (MH414573); *E*. *mediohispanicum*: Em21(MH414574), Em21 (MH414581), Em39 (MH414575), Em71 (MH414576); *E*. *nevandese*: En14 (MH414577), En10 (MH414578), En12 (MH414579), En05 (MH414580). RNA-Seq and genomic raw reads were submitted to Sequence Read Archive with the project accession number SRP149044, and the following samples accession number: *E*. *baeticum:* Ebb07 (SRR7223707), Ebb09 (SRR7223704), Ebb10 (SRR7223700), Ebb12 (SRR7223699); *E*. *mediohispanicum:* Em21(SRR7223703), Em39 (SRR7223701), Em71 (SRR7223702), Em21 (SRR7223709). *E*. *nevandese:* En14 (SRR7223710), En10 (SRR7223706), En12 (SRR7223705), En05 (SRR7223708).
