# Supplementary material for: Comparative assessment shows the reliability of chloroplast genome assembly using RNA-seq
Source: Sci Rep. 2018 Nov 27;8:17404. doi: 10.1038/s41598-018-35654-3 (PMC6258696; doi:10.1038/s41598-018-35654-3)
Supplement: Supplementary file 1 — Supplementary Material [file 41598_2018_35654_MOESM1_ESM.pdf]

Supplementary Material

Comparative assessment shows the reliability of chloroplast genome assembly using RNA-seq

Carolina Osuna-Mascaró, Rafael Rubio de Casas and Francisco Perfectti

Figure S1. Chloroplast genome map of *Erysimum baeticum*.

Figure S2. Chloroplast genome map of *Erysimum nevadense*.

Figure S3. Comparison of the borders of LSC, SSC and IR chloroplast regions among the three replicates of RNA-Seq (RNA-Seq) and the genomic libraries.

Figure S4. Mapping metrics vary with transcriptomic depth.

Table S1. Summary of the sequencing statistics.

Table S2. List of genes found in the *Erysimum cpDNA* genome.

Table S3. The length of intronic regions for the eight split genes.

Table S4. Summary of the assemblage statistics.

Table S5. Summary of characteristics of referenced-based of *Erysimum* cp genomes assembled using bwa.

Table S6. Numbers of SSR's that differed quantitatively between RNA-seq and genomic assemblages of cp genomes.

Table S7. Percentage of pairwise identity in gene comparisons across the three replicates of RNA-Seq (RNA-Seq) and including the genomic libraries (RNA-Seq + genomic).

Table S8. cp genome assembly statistics from *A.thaliana*, *E.cheiri*, *M.arvensis*, *M.sufructicosa*, *O.sativa* and *Z.mays* RNA-Seq reads using the proposed bioinformatic approach.

Figure S1. Chloroplast genome map of *Erysimum baeticum*. Genes depicted inside the circle are transcribed clockwise, and those outside are counterclockwise. Genes belonging to different functional groups are shown in different colors.

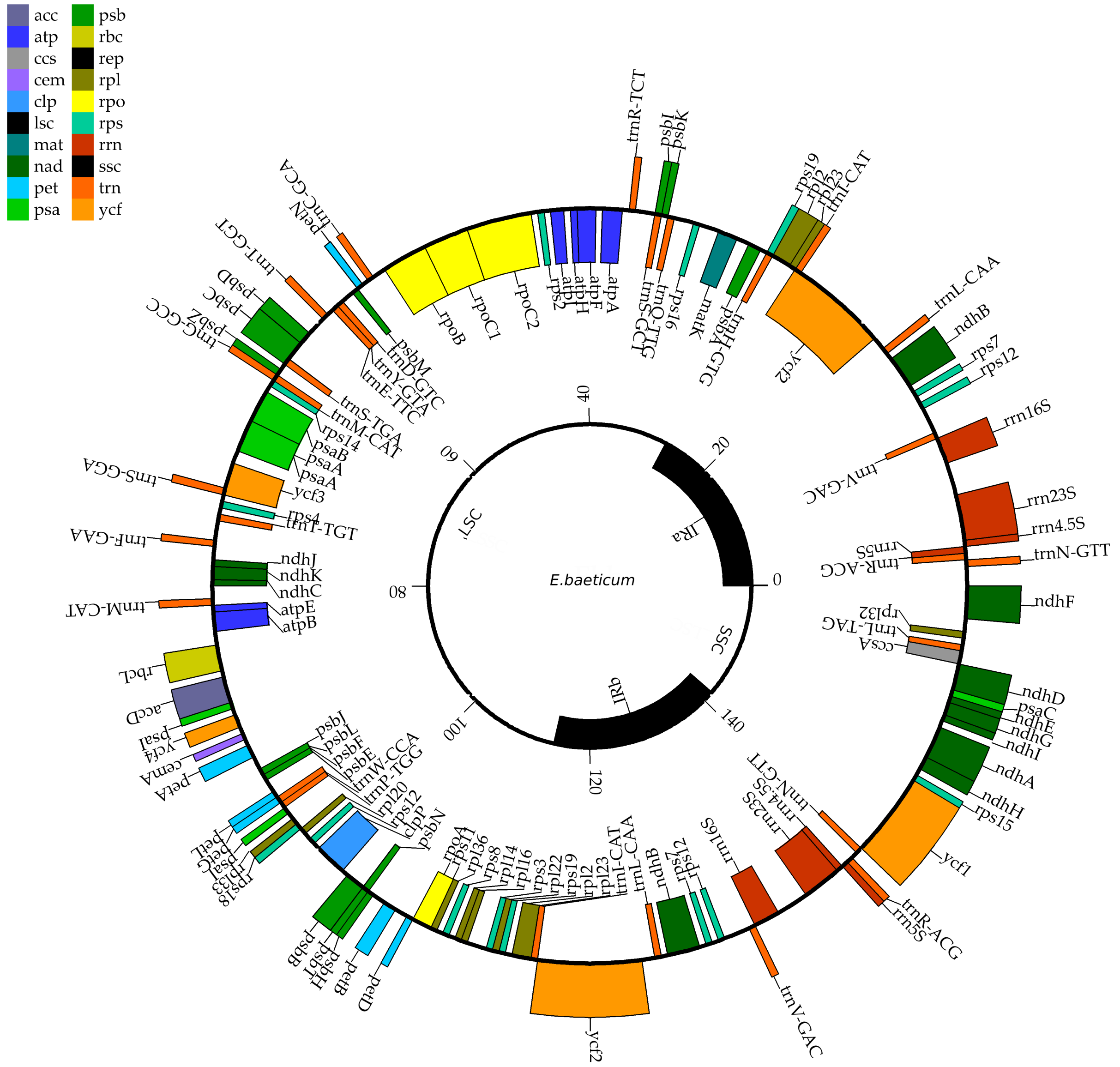

Figure S2. Chloroplast genome map of *Erysimum nevadense.* Genes depicted inside the circle are transcribed clockwise, and those outside are counterclockwise. Genes belonging to different functional groups are shown in different colors.

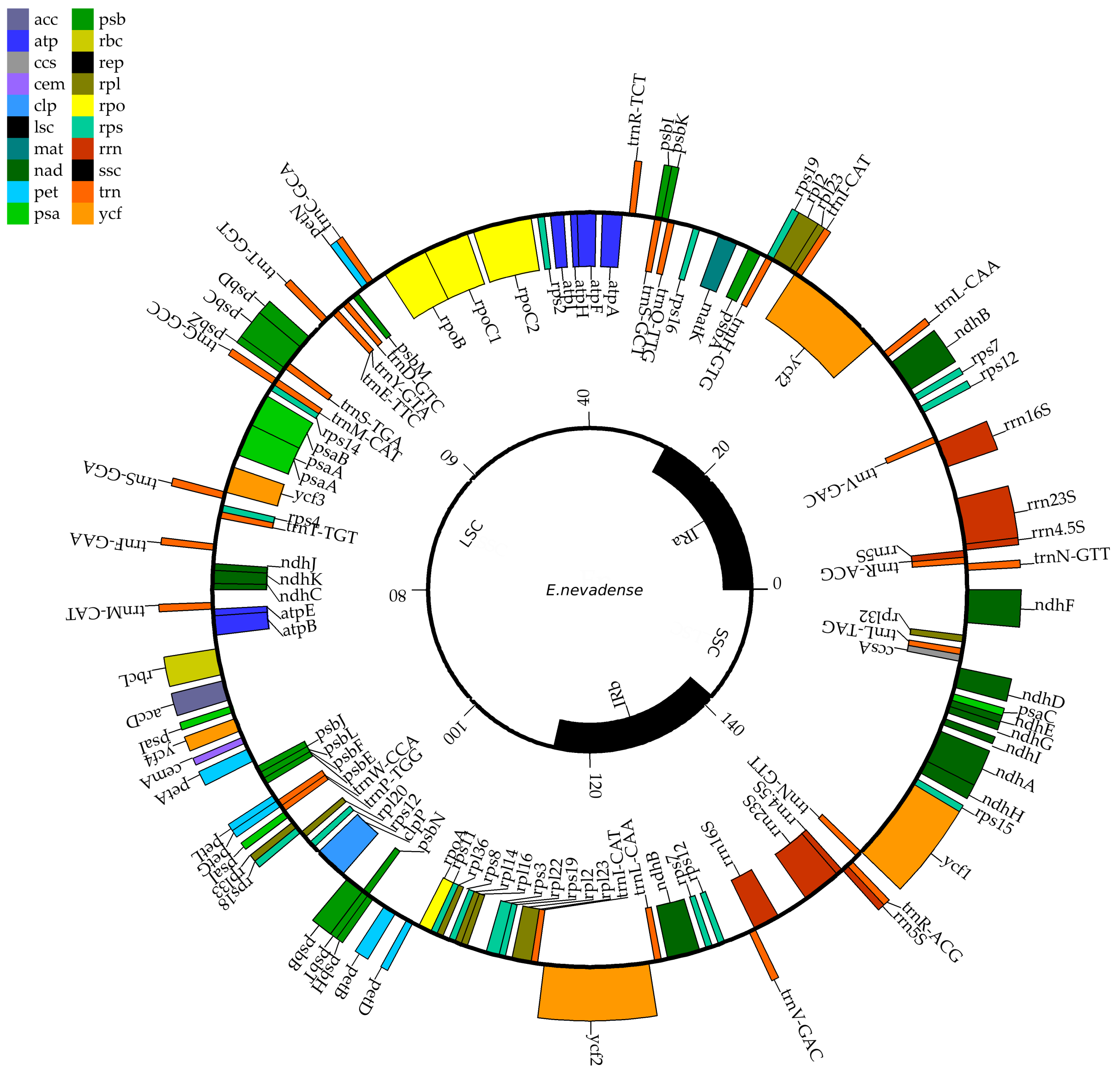

Figure S3. Comparison of the borders of LSC, SSC and IR chloroplast regions among the three replicates of RNA-Seq (RNA-Seq) and the genomic libraries. Selected genes or portions of genes are indicated by boxes.

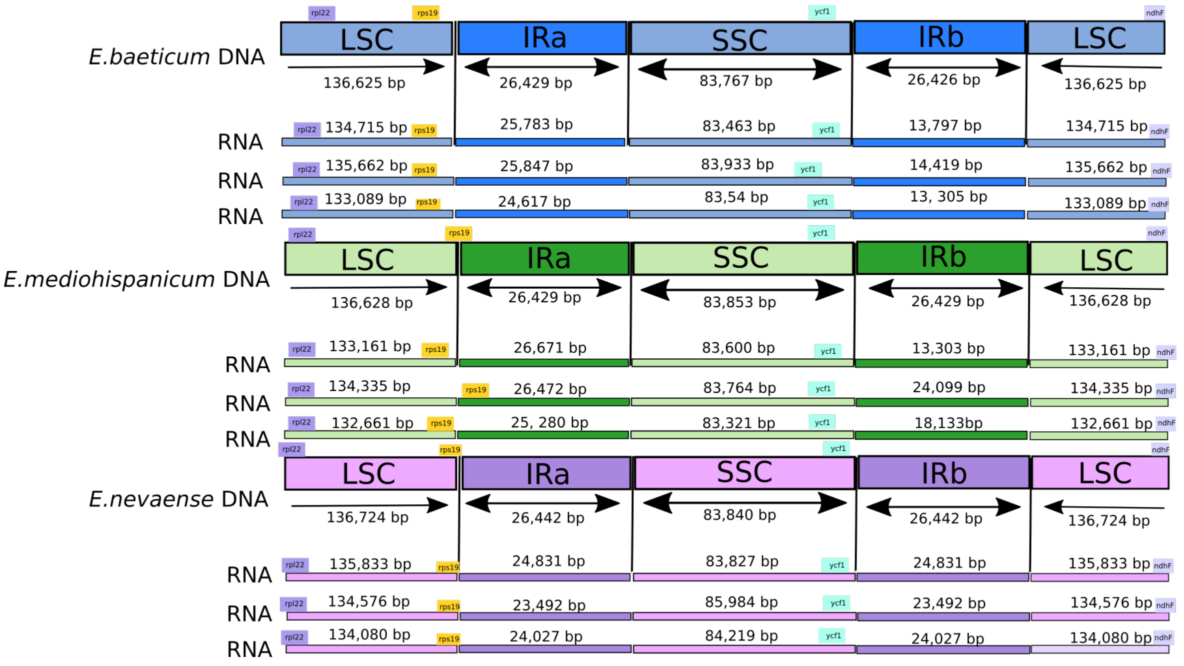

Figure S4. Mapping metrics vary with transcriptomic depth. To analyze the impact that sequencing depth has in the assemblage of transcriptome data into a complete cpDNA genome, we subsampled the RNA-seq of *E. nevadense* four times at 1 M, 5 M, 10 M, 20 M and 30 M paired reads and mapped them to the cpDNA of *E. mediohispanicum.* **(a)** Percentage of reference coverage in the consensus sequence. Fit curve was adjusted to an asymptotic grown function. **(b)** Mean confidence of the mapping based on quality scores of the reads. This is a measure of quality, with higher values indicating that a base call is more likely to be correct. Line fit was adjusted to a linear function. **(c)** Expected errors. Quality scores of the reads give the approximate number of errors that are statistically expected in mapping. The expected error value is then calculated by summing up the error rates for each base. Curve fit was adjusted to a decreasing exponential function. **(d)** Percentage of Q40 positions. Curve fit was adjusted to an asymptotic grown function

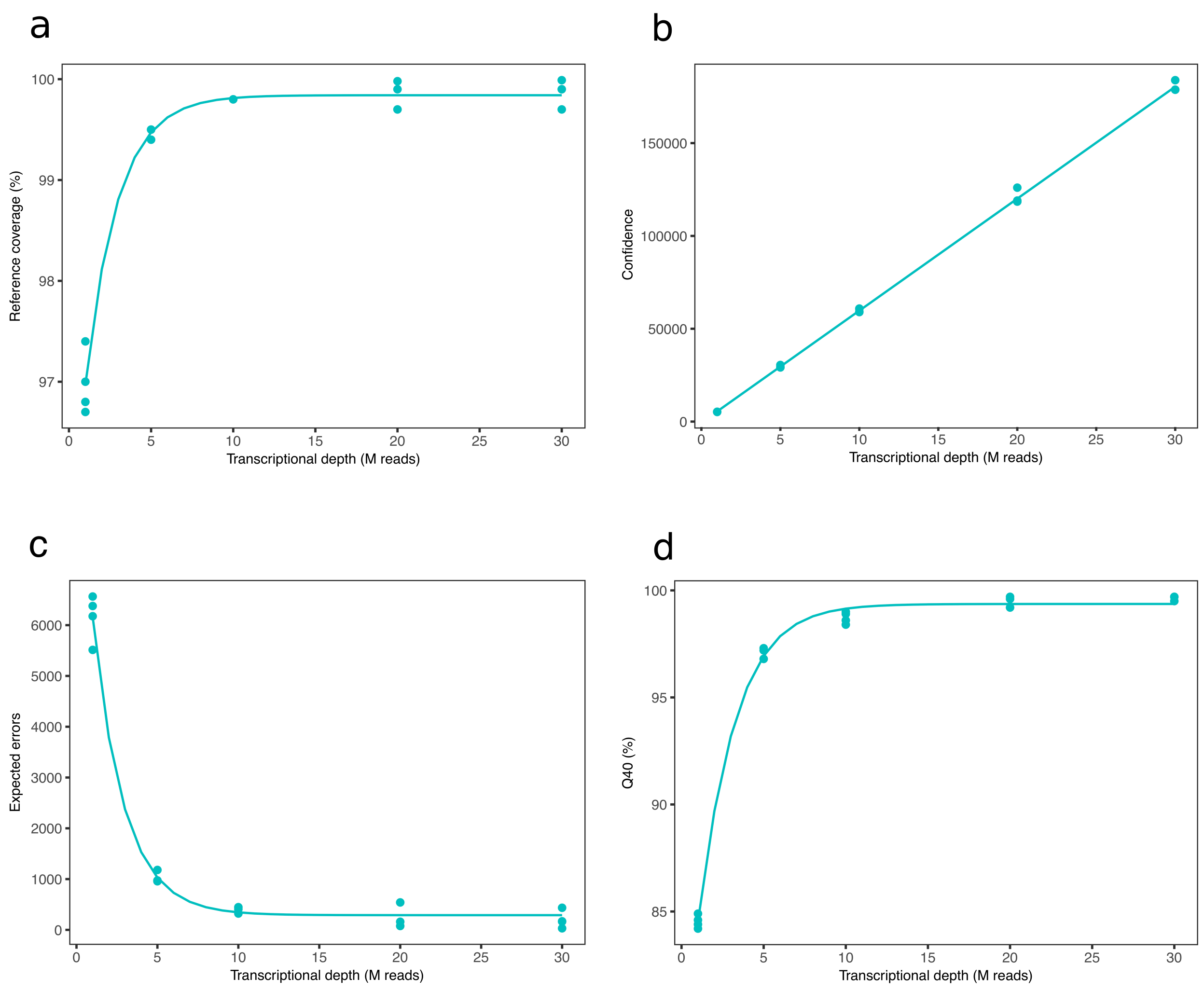

Table S1. Summary of the sequencing statistics.

Library	Species	Population code	Reads (pb)	Gb
DNA genomic	*E. baeticum*	Ebb09	403,325,778	36
	E. mediohispanicum	Em21	451,077,550	43
	E. nevadense	En14	419,863,768	37
RNA-seq	*E. baeticum*	Ebb07	68,491,616	5.1
	E. baeticum	Ebb10	134,987,506	9.9
	E. baeticum	Ebb12	136,653,914	10.1
	E. mediohispanicum	Em39	153,806,948	11.2
	E. mediohispanicum	Em21	67,787,608	4.7
	E. mediohispanicum	Em71	67,833,508	5.0
	E. nevadense	En05	147,450,544	10.8
	E. nevadense	En10	159,239,340	11.4
	E. nevadense	En12	67,164,590	5.1

Table S2. List of genes found in the *Erysimum cpDNA* genome.

**Category**	**Group of Genes**	**Name of Genes**
Photosynthesis	Subunits of ATP synthase	atpA, atpB, atpE, atpF, atpH, atpI
	Subunits of protochlorophyllide reductase	chl
	Subunits of NADH-dehydrogenase	ndhA,ndhB,ndhC, ndhE, ndhG, ndhH, ndhI, ndhJ, ndhK
	Subunits of cytochrome b/f complex	petA, petB, petD, petG, petL, petN
	Subunits of photosystem I	psaA, psaB, psaC, psaI, psaJ
	Subunits of photosystem II	psbA, psbB, psbC, psbE, psbF, psbH, psbI, psbJ, psbK, psbL, psbM, psbN, psbT, psbZ
	Subunit of rubisco	rbcl
Self replication	Large subunit of ribosome	rpl 2, 14, 16, 20, 22, 23, 32, 33, 36
	DNA dependent RNA polymerase	rpo A, B, C1, C2
	Small subunit of ribosome	rps 3, 4, 7, 8, 11, 12, 14, 15, 16, 18, 19
	rRNA Genes	rrn rrn16S, rrn23S, rrn4.5S, rrn5S
	tRNA Genes	trn trnC-GCA, trnD-GTC, trnE-TTC, trnF-GAA,trnG-GCC, trnH-GTG, trnI-CAT, trnI-CAT, trnL-CAA, trnL-CAA, trnL-TAG, trnM-CAT, trnM-CAT, trnN-GTT, trnN-GTT, trnP-TGG, trnQ-TTG, trnR-ACG, trnR-ACG, trnR-TCT, trnS-GCT, trnS-GGA, trnS-TGA, trnT-GGT, trnT-TGT, trnV-GAC, trnV-GAC, trnW-CCA, trnY-GTA
Unknown function	Conserved open reading frames	ycf1, ycf2, ycf3, ycf4, ycf5
Other	Subunit of Acetyl-CoA-carboxylase	aacD
	c-type cytochrom synthesis gene	ccsA
	Envelop membrane protein	cemA
	Protease	clpP
	Translational initiation factor	infA
	Maturase	matK
	Elongation factor	tuf

Table S3. Length of the intronic regions for the eight split genes.

Gene	Intron length
ndhB	685
rpl2	682
atpF	723
rpoC1	799
psaA	30
ycf3	710
clpP	873
ndhA	1070

Table S4. Summary of the assembly statistics: Number of bases (the number of bases assembled), mean coverage (the mean of the coverage for each base in the consensus sequence), and the pairwise identity (the average % of identity -considering ambiguity characters- over the alignment, with 100 indicating identical agreement).

Taxon	Population code	Library	Number of bases (bp)	Mean coverage	Pairwise identity (%)
E. baeticum	Ebb07	DNA genomic	3,742,777	4,513	97.3
	Ebb09	RNA-seq	14,099,423	12,827.6	99.7
	Ebb10	RNA-seq	9,995,492	12,171	96.4
	Ebb12	RNA-seq	9,692,811	11,468	92.9
E. mediohispanicum	Em21	DNA genomic	43,187,499	39,097.9	98.9
	Em21	RNA-seq	3,257,794	5,454.6	97.1
	Em71	RNA-seq	5,441,636	8,809.3	95.8
	Em39	RNA-seq	13,294,086	17,269.6	95.8
E. nevadense	En14	DNA genomic	56,955,629	52,891.8	98.6
	En05	RNA-seq	9,342,246	12,909.8	96.1
	En10	RNA-seq	12,271,273	17,956.2	96.1
	En12	RNA-seq	5,627,498	7,805.9	96.4

Table S5. Summary of characteristics of referenced-based of *Erysimum* cp genomes assembly using BWA. Type of library (genomic DNA or RNA-Seq library), length of the cp genome (bp), number of assembled reads, length of the two inverted repeats (IR a and the IR b), length of the small single copy (SSC) and of the large single copy (LSC) regions, and GC% content.

Taxon	Population code	Type of library	Lenght (bp)	Assembled reads	IR a (bp)	SSC (pb)	IR b (bp)	LSC (bp)	GC (%)
E. baeticum	Ebb09	Genomic DNA	154,581	983,811	26,429	83,767	26,426	136,625	36.5
	Ebb07	RNA-Seq libraries	154,691	3,395,688	26,429	95,235	13,675	134,715	37.5
	Ebb10	RNA-Seq libraries	154,564	8,836,821	25,702	95,456	14,444	135,662	36.5
	Ebb12	RNA-Seq libraries	154,761	9,126,450	24,987	95,167	13,304	133, 088	36.5
E. mediohispanicum	Em21	Genomic DNA	154,599	1,414,714	26,429	98,752	13,303	136,628	36.3
	Em71	RNA-Seq libraries	154,788	4,999,161	24,617	98,165	14,919	133,088	36.7
	Em39	RNA-Seq libraries	154,687	11,730,858	26,356	98,248	18,345	133, 161	36.7
	Em21	RNA-Seq libraries	154,251	1,988,198	25,280	89,248	18, 333	129,067	36.7
E. nevadense	En14	Genomic DNA	154,661	1,554,542	26,442	83,840	26,442	132,661	36.6
	En05	RNA-Seq libraries	153, 467	10,925,889	25,634	83,357	24, 833	136,722	36.7
	En10	RNA-Seq libraries	154,832	8,326,981	25,900	83,212	22, 256	136,737	36.6
	En12	RNA-Seq libraries	154,701	4,751,517	25,760	80,119	23, 027	135,755	36.6

Table S6. Numbers of SSR's that differed quantitatively between RNA-seq and genomic assemblages of cp genomes.

Taxon	Population code	Library	A	C	G	T	AT	TA	TTA	ATAG	CAAA	TAAA	TTAA	ATTAG	ATAGAA
E. baeticum	Ebb07	DNA genomic	23	2	1	24	11	8	1	1	2	1	1	1	1
	Ebb09	RNA-seq	18	2	1	23	8	5	1	0	1	2	1	1	1
	Ebb10	RNA-seq	17	2	1	20	11	5	1	0	1	2	1	1	1
	Ebb12	RNA-seq	17	2	1	21	11	5	1	0	1	2	1	1	1
E. mediohispanicum	Em21	DNA genomic	19	2	1	27	11	6	1	1	1	2	1	1	1
	Em21	RNA-seq	16	1	0	21	9	5	1	0	1	1	1	1	0
	Em71	RNA-seq	16	2	1	21	11	5	1	0	0	1	2	1	1
	Em39	RNA-seq	17	2	1	21	11	5	1	0	1	2	1	1	1
E. nevadense	En14	DNA genomic	24	2	1	25	12	6	2	1	2	1	1	2	0
	En05	RNA-seq	18	2	1	21	11	5	1	0	1	2	1	1	1
	En10	RNA-seq	17	2	1	21	11	5	1	0	1	2	1	1	1
	En12	RNA-seq	17	2	1	18	8	3	1	0	1	2	1	1	1

Table S7. Percentage of pairwise identity in gene comparisons across the three replicates of RNA-Seq (RNA-Seq) and including the genomic libraries (RNA-Seq + genomic).

**Category**	**Gen**	***E. nevadense***		***E. mediohispanicum***		***E. baeticum***	
		**RNA-Seq**	**RNA-Seq + genomic**	**RNA-Seq**	**RNA-Seq + genomic**	**RNA-Seq**	**RNA-Seq + genomic**
Photosynthesis genes	rbcl	100	99.9	100	100	100	100
	psaA	100	99.9	100	100	99.9	99.8
	psbA	100	99.9	99.9	99.9	99.98	99.97
	ndhK	99.9	99.8	99.8	99.9	100	100
	atpA	100	99.8	100	100	99.9	99.9
	atpH	99.2	99.4	100	100	99.6	99.5
Self replication	rpoA	100	99.8	100	99.97	99.9	99.9
	rps3	99.97	99.9	99.97	99.9	99.8	99.9
	rrn16S	100	99.8	100	100	99.9	99.9
	trnH	99.9	100	99.96	99.97	99.9	99.9
Other Genes	matK	100	100	100	100	100	100
	ycf2	100	99.9	100	100	99.9	99.9

Table S8. cp genome assembly statistics from *A.thaliana*, *E.cheiri*, *M.arvensis*, *M.sufructicosa*, *O.sativa* and *Z.mays* RNA-Seq reads using the proposed bioinformatic approach.

**Species**	**RNA-Seq library**	**Tissue**	**Genbank chloroplast genome reference**	**Consensus lenght (bp)**	**Confidence mean**	**Q20 (%)**	**Q30 (%)**	**Q40 (%)**	**Assembled reads**	**Pairwise identity (%)**	**Mean coverage**	**Ref-Seq (%)**
Arabidopsis thaliana	SRR6676021	leaf	*A. thaliana* NC_001666 (140,384 bp)	150,757	1,711.1	80.7	77.6	71.6	608,988	89.3	315.1	96.0
Arabidopsis thaliana	SRR6757372	leaf	*A. thaliana* NC_001666 (140,384 bp)	138,157	24,592.1	85.4	83.2	80.9	6,618,957	99.8	1184.1	91.4
Erysimum cheiri	SRR5195369	petal	*E. mediohispanicum* MH414570 (154,599 bp)	79,757	1,585.2	66.8	64.8	57.0	54,362	76.2	136.1	86.1
Moricandia arvensis	SRR4296231	leaf	*Brassica napus* GQ861354 (152,860 bp)	139,300	4,030.8	67.9	66.9	60.7	361,750	94.0	353.9	80.6
Moricandia suffruticosa	SRR4296233	leaf	*Brassica napus* GQ861354 (152,860 bp)	135,131	2,834.7	63.7	62.1	54.5	231,408	88.4	225.0	76.8
Oryza sativa	SRR7079258	seedling	*O. sativa* NC_001320 (134,525 bp)	103,417	514.7	49.5	43.0	39.5	79,647	93.7	114.6	79.8
Zea mays	ERR1407273	seedling root	*Z. mays* NC_001666 (140,384 bp)	112,606	1,741.5	68.3	66.7	61.9	58,591	71.4	211.1	78.7
